# Correlation between CT-determined emphysema and the severity of acute exacerbation of chronic obstructive pulmonary disease: a cross-sectional study

**DOI:** 10.3389/fmed.2026.1825493

**Published:** 2026-07-14

**Authors:** Cheng Yang, Guan-Xia Cao, Hai-Hui Wu, Han-Hua Zeng, Juan Huang, Hua-Gen Zhang

**Affiliations:** 1 Department of Pulmonary and Critical Care Medicine, Meizhou People’s Hospital, Meizhou, Guangdong, China; 2 First School of Clinical Medicine, Guangdong Medical University, Zhanjiang, Guangdong, China; 3 Department of Radiology, Meizhou People’s Hospital, Meizhou, Guangdong, China

**Keywords:** acute exacerbation, chronic obstructive pulmonary disease, CT scan, emphysema, health-related quality of life, lung function

## Abstract

**Background:**

The high prevalence of chronic obstructive pulmonary disease (COPD) poses a significant social burden. Emphysema is a basic pathological change associated with poor lung function in stable COPD. However, few studies have focused on the potential role of emphysema in acute exacerbation of COPD (AECOPD). The current study aimed to explore the correlation between CT-determined emphysema and other main physiological parameters of severe AECOPD.

**Methods:**

In total 90 hospitalized patients with AECOPD participated in this cross-sectional study. All of them underwent chest CT scans and lung function tests as planned. Emphysema was defined as the percentage of low attenuation areas (LAA) below −950 Hounsfield Units (%LAA_−950_) on inspiratory CT scans. Health-Related Quality of Life (HRQoL) was assessed using four questionnaires, including the St. George’s Respiratory Questionnaire (SGRQ), modified Medical Research Council dyspnea scale, COPD Assessment Test, and EXAcerbation of Chronic Pulmonary Disease Tool (EXACT). Qualified data from 73 patients were included in the analysis.

**Results:**

%LAA_−950_ was significantly correlated with spirometry parameters and HRQoL scores. The SGRQ and EXACT scores were significantly positively correlated with %LAA_−950_ (*ρ* = 0.399, *p* < 0.001; *ρ* = 0.370, *p =* 0.001, respectively). The ratio of the forced expiratory volume in the first 1 s to the forced vital capacity of the lungs and the percentage of predicted forced expiratory volume in first 1 s, which are functional indicators of airflow limitation, were significantly negatively correlated with %LAA_−950_ (*ρ* = −0.735, *p* < 0.00; *ρ* = −0.602, *p* < 0.001, respectively). Based on the stepwise multiple linear regression model showed that FEV_1_/FVC and age could be predictors of %LAA_−950_ (*R*^2^ = 0.592, *F* = 50.762, *p* < 0.001).

**Conclusion:**

In patients with severe AECOPD, CT-determined emphysema is associated with decreased lung function and poor HRQoL.

**Trial registration:**

The study was registered on the Chinese Clinical Trial Registry (registration number: ChiCTR2000033101).

## Introduction

Chronic Obstructive Pulmonary Disease (COPD) is a heterogeneous disease characterized by chronic respiratory symptoms (such as dyspnea, cough, sputum production, and acute exacerbation) caused by airway (bronchitis, bronchiolitis) and/or alveolar abnormalities (emphysema), resulting in persistent and frequently progressive airflow limitation (1). COPD has become a major global public health issue, significantly affecting the quality of life and survival prognosis. Further, it is the third leading cause of mortality worldwide, with 90% of deaths occurring in low- and middle-income countries (2).

Emphysema is a basic pathological change in COPD, mainly characterized by the destruction of the alveoli and loss of lung elasticity (3). High-resolution CT is an ideal method for detecting emphysema and is widely used to determine the extent of emphysema and small-airway involvement in COPD (4). The CT threshold of normal lungs at the end of deep inspiration is approximately −850 Hounsfield Units (HU). Air-containing spaces replace normal lung tissues. Thus, the inspiratory CT threshold of emphysematous lungs approaches −1,000 HU (5). The low attenuation areas below −950 HU (LAA_−950_) on inspiratory CT represent pathological emphysema (6), which was quantified by the percentage of LAA_−950_ to the total lung capacity.

Emphysema is associated with decreased lung function (7), and poor quality of life assessed using the St George’s Respiratory Questionnaire (SGRQ) (8). Further, it is an independent risk factor for frequent acute exacerbations of COPD (AECOPD) (9). Collectively, emphysema is well established as a key determinant of clinical outcomes in stable COPD, including exacerbation risk, disease progression, and mortality (1).

AECOPD is a condition characterized by dyspnea worsening and/or increased cough and sputum production within 14 days, often associated with elevated local and systemic inflammation caused by airway infections, contamination, or other lung injury (1). Acute exacerbation is the main cause of morbidity and mortality in COPD (10). However, currently available tools for assessing disease severity during acute exacerbations have notable limitations: symptom assessments rely on patients’ responses to questionnaires and are therefore subjective (11), while qualified pulmonary function test results are frequently unobtainable in the acute setting (12). In contrast, CT offers a more objective and readily available assessment. Therefore, we hypothesized that CT-determined emphysema at COPD exacerbation is related to its severity as determined by lung function and symptom burden.

This research aimed to explore the correlation between CT-determined emphysema and functional indicators, including lung function parameters, Health-Related Quality of Life (HRQoL), and laboratory tests results, which reflect disease severity, by analyzing a cross-sectional study on patients with AECOPD requiring hospitalization.

## Materials and methods

### Participants

Ninety patients diagnosed with AECOPD requiring hospitalization at Meizhou People’s Hospital in Guangdong, China, from May 2020 to January 2021 were screened in the cross-sectional study. Inclusion criteria were as follows: post-bronchodilator FEV_1_/FVC ratio less than 0.7, diagnosis of AECOPD (10), and written informed consent obtained. Exclusion criteria were as follows: contraindications for lung function test, other respiratory diseases besides COPD (sarcoidosis, active tuberculosis, pulmonary fibrosis, cystic fibrosis, lung cancer, etc.), or conditions deemed by investigators to significantly affect clinical evaluation, or inability to read and understand Mandarin.

In total, 17 participants were excluded, including 11 patients who refused to undergo lung function tests or did not obtain qualified lung function test results, 5 who were diagnosed with lung diseases other than COPD, and one did not undergo eligible chest CT scans. Finally, 73 participants were included in the analysis.

### Study protocol

Electrocardiogram and assessment of C-reactive protein (CRP) levels, complete blood count, and four coagulation items (prothrombin time, activated partial thromboplastin time, thrombin time, and fibrinogen levels) were performed on the first day of admission. The CT scans and HRQoL (13) assessments using SGRQ, modified Medical Research Council dyspnea scale (mMRC), COPD Assessment Test (CAT), and EXAcerbation of Chronic Pulmonary Disease Tool (EXACT) were completed on the next day. The lung function test was accomplished within 12–48 h after the HRQoL assessment. The study process did not intervene with the clinicians’ decisions. The pulmonary function technologists, testing technicians, and radiologists were blinded to the other clinical data of the patients. Before including the first participant, the study was registered on the Chinese Clinical Trial Registry (registration number: ChiCTR2000033101).

### CT scan and quantitative emphysema assessment

**Figure 1:**
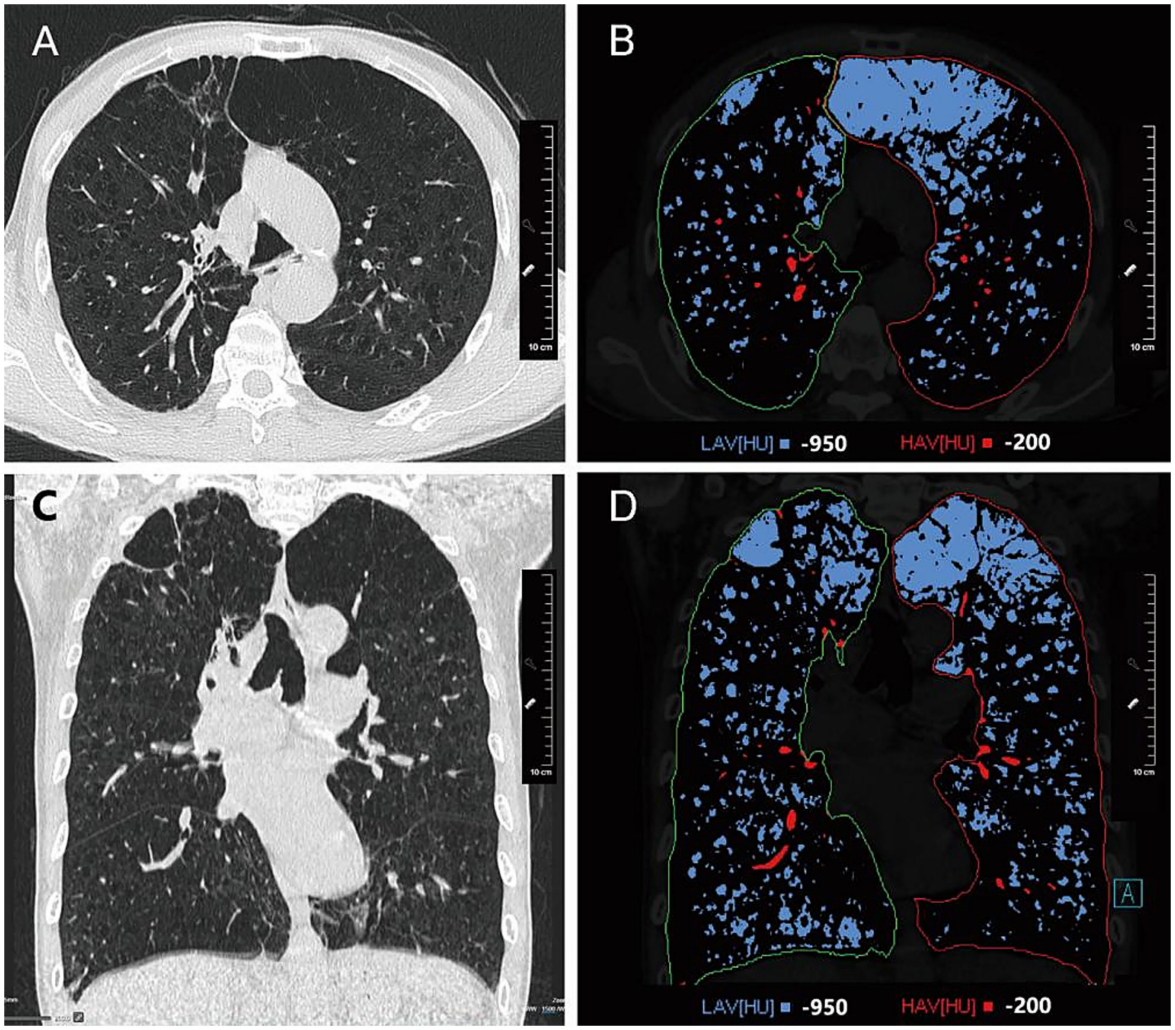
CT quantification of emphysema using the %LAA_−950_ index. The left column shows lung-window CT images in the axial **(A)** and coronal **(C)** planes. The right column shows 3D-reconstructed CT images in the axial **(B)** and coronal **(D)** planes. The blue areas represent lung regions with low attenuation areas below −950 HU (indicative of emphysema), and the red areas represent high attenuation areas above −200 HU. In this subject, the %LAA_−950_ was 36%. %LAA_−950_, the ratio of low attenuation areas below −950 HU to total lung volume measured by CT scan.

All participants underwent high-resolution CT scan of the chest using German Siemens (SOMATOM Force, Siemens Healthineers, Forchheim, Germany). The scanning parameters were set as follows: slice thickness, 5 mm; tube voltage, 120 kV with a PITCH of 1.2; automatic tube current modulation; rotation time, 0.5 s; and matrix, 512 × 512. The participants were trained to deeply inhale and hold their breath according to the operator’s instruction before the procedure. The patients were placed in the supine position with both hands raised to the head and, with head entering first. At the end of the deep inhalation, the patients held their breath and underwent a full scan from the tip to the bottom of the lungs. For further analysis, all images were stored in the DICOM format and transmitted to a dedicated workstation (see Figure 1). The total lung capacity (TLC_CT_) was defined as the whole lung volume measured on CT scan, and the emphysema area was defined as low attenuation areas below −950 HU (LAA_−950_). The ratio of LAA_−950_ to TLC_CT_ (%LAA_−950_) was used to quantify emphysema. A radiologist performed quantitative analysis of emphysema extent using syngo. CT Pulmo 3D version VB40 (SOMATOM Force, Siemens Healthineers, Forchheim, Germany), which automatically calculated the LAA_−950_, TLC_CT_ and %LAA_−950_ (14).

### Pulmonary function test

According to the European Respiratory Association/American Thoracic Association guidelines (16), pulmonary function tests were performed using microQuark PFT (Cosmed, Rome, Italy). After 4–6 tidal breaths, the participants fully inhaled to the TLC level, and then exhaled for at least 6 s with maximum effort and speed to the residual volume (RV) level. Twenty minutes after inhaling 400 μg of Salbutamol (GlaxoSmithKline, London, the United Kingdom), spirometry was repeated. Within 1 day, the pre- or postbronchodilator spirometry parameters could be checked up to 8 times, and the postbronchodilator parameters were adopted in the statistical analysis.

### Health-related quality of life

HRQoL (13) was assessed using the mMRC, SGRQ, CAT, EXACT. The mMRC, with scores of 0–4, is applied to investigate the degree of dyspnea in patients with COPD (17). The SGRQ is a widely used questionnaire, with scores of 0–100, designed to assess airway diseases that lead to a decline in quality of life (18). The CAT is a validated questionnaire comprising eight items used to measure and quantify the symptom on the health status of patients with COPD (10). EXACT is a tool utilized to directly evaluate self-reported exacerbation symptoms in patients with chronic airway diseases and to perform standardized evaluation of their condition (19). The participants were instructed to select the description that is most consistent with their symptoms or their level of agreement with each item. One of the authors was always present to provide explanations to the participants but did not offer any advice. In all four questionnaires, a higher score or grade indicated a poorer quality of life.

### Statistical analyses

All data were processed and analyzed using the Statistical Package for the Social Sciences software version 22.0 (IBM Corporation, Armonk, NY, United States). All results were presented with descriptive data. As appropriate, the findings were expressed as the mean ± standard deviation or median (interquartile range [IQR]: 25–75). According to the data distribution, the *t*-test or the Wilcoxon rank-sum test was used to test the differences in parameters between patients who were included and those who were excluded. The Spearman’s rank correlation coefficient was utilized to assess the correlation of CT-determined emphysema with lung function indicators, HRQoL assessment scores, and laboratory findings. The correlation coefficient was expressed as *ρ*.

The indicators of significant correlation were used as the candidate covariates for the subsequent multiple linear regression analysis. The stepwise regression method was used to conduct multiple linear regression analysis to further explore the correlation between CT-determined emphysema and other variables. A bilateral *p* value of < 0.05 indicated statistically significant differences. The Bonferroni correction was used to redetermine the alpha level for the correlation analyses to adjust for multiple testing. Conversely, the correction was not applied to the regression analyses, as it would reduce statistical power and thereby limit the detection of potentially important variables.

## Results

### Characteristics of the participants

There was no statistically significant difference in terms of characteristics between the participants who were included and those who were excluded (*p* > 0.05). Of the 73 patients who were included, 72 (97.8%) were men. The average age of the patients was 68.2 ± 6.2 years, and the median body mass index (BMI) was 19.2 (IQR: 25–75, 17.5–21.9) kg/m^2^. Table 1 shows the characteristics of the participants. Based on clinical judgment, airway specimens (sputum or bronchoalveolar lavage fluid) were collected for culture, which yielded positive results in four patients: two grew *Pseudomonas aeruginosa* and the other two grew *Klebsiella pneumoniae*.

**Table 1:** Comparison between included and excluded patients.

Characteristics	Included patients (*n* = 73)	Excluded patients (*n* = 17)	Test statistic	*p*-value
Demographic factor				
Age (year)	68.2 ± 6.2	68.4 ± 5.9	−0.152	0.880
Sex, male (%) ^a^	72 (97.8%)	16 (94.1%)	−−	0.344
BMI (kg/m^2^)	19.2 (17.5–21.9)	20.1 (18.4–23.7)	−1.289	0.198
Amount of smoking				
Pack-years	56.1 ± 30.2	50.5 ± 26.6	0.695	0.489
Chronic bronchitis, yes (%)	52 (71.2%)	11 (64.7%)	0.280	0.597
Quality of life				
CAT score	13.6 ± 8.0	14.7 ± 9.7	−0.496	0.621
mMRC grade	2.0 (1.0–2.0)	2.0 (1.0–3.0)	−1.141	0.254
SGRQ score	49.0 (38.0–63.0)	60.7 (32.9–71.9)	−1.155	0.248
EXACT score	15.6 ± 8.7	18.2 ± 8.7	−1.125	0.264
Complete blood count test				
White blood cell (10^9^/L)	7.6 (6.4–9.7)	8.4 (6.7–10.2)	−0.918	0.359
Neutrophil (10^9^/L)	5.1 (4.1–7.0)	5.9 (4.5–8.1)	−1.124	0.261
Lymphocyte (10^9^/L)	1.5 (1.2–1.8)	1.4 (1.0–1.7)	1.069	0.285
Eosinophil (10^9^/L)	0.2 (0.1–0.3)	0.2 (0.1–0.3)	0.893	0.372
Red blood cell (10^12^/L)	4.7 (4.4–5.0)	4.5 (4.4–4.8)	0.907	0.364
Hemoglobin (g/L)	142.5 ± 13.7	144.1 ± 12.0	−0.433	0.666
Arterial blood gas test				
PaCO_2_ (mmHg)	40.3 ± 6.9	42.5 ± 10.8	−0.802	0.432
Lactic acid (mmol/L)	1.3 (1.1–1.7)	1.2 (0.9–1.7)	0.475	0.634
Other laboratory findings				
C-reactive protein (mg/L)	2.8 (1.3–14.6)	3.1 (1.6–35.5)	−0.629	0.529
Albumin (g/L)	37.5 ± 3.8	37.6 ± 3.7	−0.059	0.953
Blood urea nitrogen (mmol/L)	5.7 (4.3–7.3)	5.3 (4.2–7.2)	0.593	0.553
Creatinine (μmol/L)	70.3 (60.0–80.2)	72.0 (62.6–90.8)	−0.948	0.343
Fbrinogen (g/L)	3.6 (3.1–4.6)	3.8 (2.9–5.0)	−0.356	0.722
Comorbidity, yes (%)	33 (45.2%)	10 (58.8%)	0.552	0.458
Left ventricular ejection fraction (%)	65.0 (63.0–67.0)	65.0 (63.5–66.0)	−0.145	0.885

^a^Fisher’s exact test. Results are expressed as mean ± SD or median (IQR: 25–75) as appropriate.

BMI, body mass index; CAT, Chronic Obstructive Pulmonary Disease Assessment Test; mMRC, modified Medical Research Council dyspnea scale; SGRQ, St George’s Respiratory Questionnaire; EXACT, EXAcerbation of chronic pulmonary disease Tool; PaCO_2_, partial pressure of carbon dioxide in artery.

The mean TLC_CT_, LAA_−950_, and %LAA_−950_ were 5603.6 ± 953.1 mL, 1195.3 ± 808.2 mL, and 20.18% ± 12.08%, respectively. The median reduced forced expiratory flow between 25 and 75% of vital capacity percent predicted (FEF_25–75%_), which reflects small-airway function was 0.42 L/s (IQR: 25–75, 0.31–0.62 L/s). The mean percentage of predicted forced expiratory volume in 1 s (FEV_1_% predicted) was 42.3% ± 15.9%. Table 2 shows the parameters of postbronchodilator lung function and CT -determined emphysema in 73 patients who were included.

**Table 2:** The parameters of postbronchodilator lung function and CT-determined emphysema in included patients.

Characteristics	*n* = 73
Spirometric parameters	
FVC (L)	2.26 ± 0.54
FVC % predicted	69.5 ± 15.6
FEV_1_(L)	0.97 (0.73–1.37)
FEV_1_% predicted	42.3 ± 15.9
FEV_1_ ≥ 80%predicted, *n* (%)	2 (2.7%)
50% ≤ FEV_1_ < 80%predicted, *n* (%)	21 (28.8%)
30 ≤ FEV_1_ < 50%predicted, *n* (%)	33 (45.2%)
FEV_1_ < 30%predicted, *n* (%)	17 (23.3%)
FEV_1_/FVC (%)	44.4 (40.6–51.9)
PEF (L/s)	2.68 ± 1.13
FEF_25%–75%_ (L/s)	0.42 (0.31–0.62)
CT-determined emphysema	
TLC_CT_ (mL)	5603.6 ± 953.1
LAA_−950_ (mL)	1195.3 ± 808.2
%LAA_−950_ (%)	20.18 ± 12.08

Results are expressed as mean ± SD or median (IQR: 25–75) as appropriate.

FVC, forced vital capacity; FEV_1_, forced expiratory volume in the first 1 s; FEV_1_% predicted, the percentage of predicted forced expiratory volume in 1 s; FVC % predicted, the percentage of predicted forced vital capacity; PEF, peak expiratory flow; FEF_25–75%_, forced expiratory flow at 25 to 75% of the FVC; TLC_CT_, total lung volume measured by CT scan; LAA_−950_, low attenuation areas below −950 HU; %LAA_−950_, the ratio of LAA_−950_ to TLC_CT_.

### Correlation between %LAA_−950_ and other parameters

**Figure 2:**
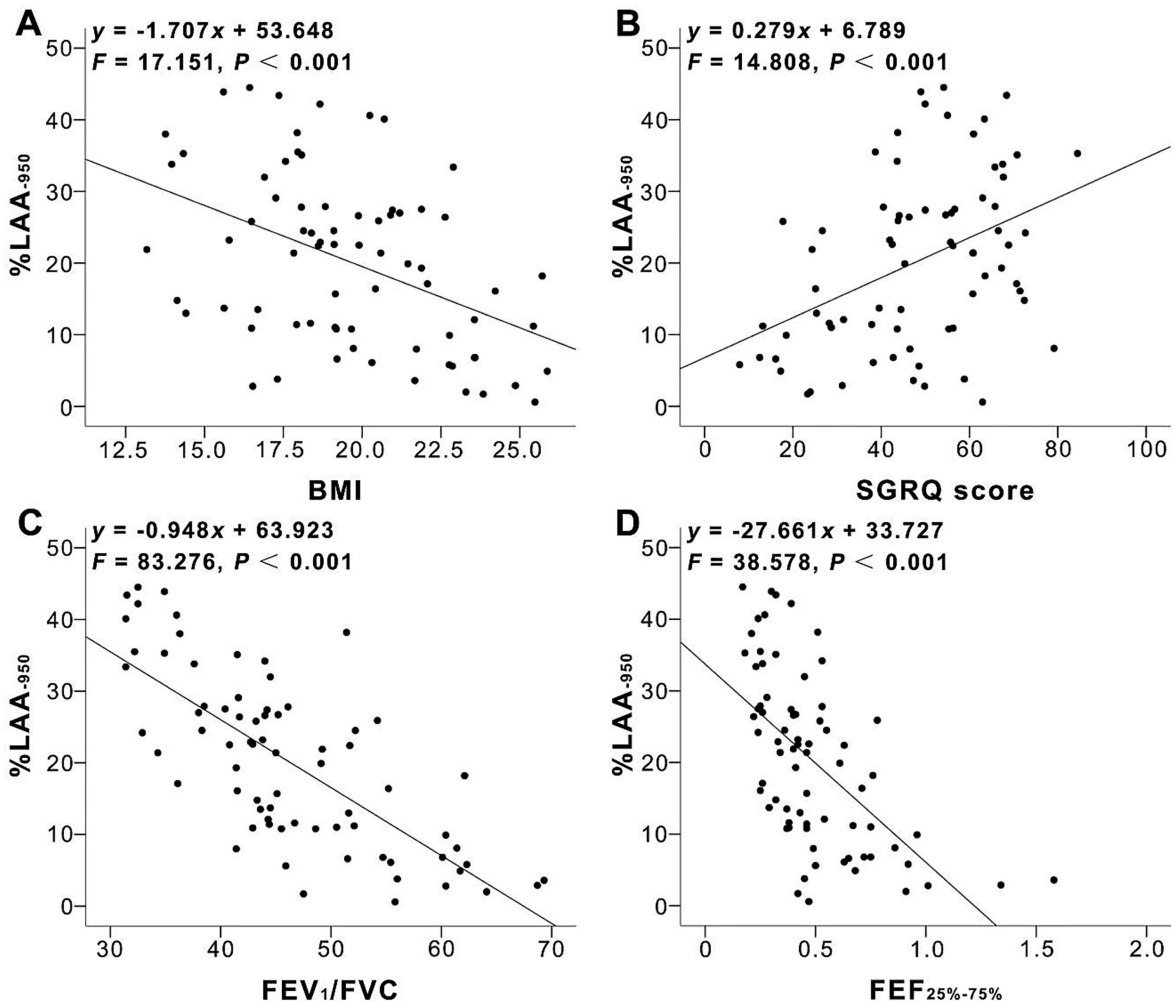
Correlation of %LAA_−950_ with BMI, SGRQ score, FEV_1_/FVC and FEF_25%–75%_. Notes: %LAA_−950_ is significantly correlated with BMI **(A)**, SGRQ score **(B)**, FEV_1_/FVC **(C)** and FEF_25–75%_ **(D)**. %LAA_−950_, the ratio of low attenuation areas below −950 HU to total lung volume measured by CT scan; BMI, body mass index; SGRQ, St George’s Respiratory Questionnaire; FVC, forced vital capacity; FEV_1_, forced expiratory volume in the first 1 s; FEF_25–75%_, forced expiratory flow at 25 to 75% of the FVC.

Table 3 depicts the correlation of %LAA_−950_ with lung function parameters, HRQoL assessment scores, and laboratory test results. At baseline, age and presence of chronic bronchitis were weakly positively correlated with %LAA_−950_ (*ρ* = 0.248, *p* = 0.035, and *ρ* = 0.278, *p* = 0.017, respectively). BMI was significantly negatively correlated with %LAA_−950_ (*ρ* = −0.439, *p* < 0.001) (Figure 2). Meanwhile, the amount of smoking was not significantly correlated with %LAA_−950_.

**Table 3:** Correlation between %LAA_−950_ and other parameters.

Characteristics	*ρ*	95%CI	*P*-value
Age	0.248	0.008 to 0.464	0.035^a^
Sex, male (%)	0.201	0.194 to 0.388	0.088
BMI (kg/m^2^)	−0.439	−0.619 to −0.225	<0.001^a^
Amount of smoking (pack-years)	0.150	−0.089 to 0.384	0.206
Chronic bronchitis (yes)	0.278	0.045 to 0.487	0.017^a^
CAT score	0.340	0.141 to 0.524	0.003^a^
mMRC grade	0.362	0.144 to 0.550	0.002^a^
EXACT score	0.370	0.137 to 0.542	0.001^a^
SGRQ score	0.399	0.177 to 0.551	<0.001^a^
White blood cell (10^9^/L)	−0.019	−0.256 to 0.225	0.871
Eosinophil (10^9^/L)	−0.069	−0.293 to 0.184	0.560
PaCO_2_ (mmHg)	0.330	0.105 to 0.530	0.004^a^
C-reactive protein (mg/L)	−0.084	−0.287 to 0.155	0.482
Albumin (g/L)	−0.292	−0.497 to −0.047	0.012^a^
FVC (L)	−0.372	−0.570 to −0.145	0.001^a^
FVC % predicted	−0.353	−0.549 to −0.120	0.002^a^
FEV_1_(L)	−0.626	−0.751 to −0.456	<0.001^a^
FEV_1_% predicted	−0.602	−0.742 to −0.407	<0.001^a^
FEV_1_/FVC (%)	−0.735	−0.833 to −0.580	<0.001^a^
PEF (L/s)	−0.465	−0.615 to −0.269	<0.001^a^
FEF_25%–75%_ (L/s)	−0.639	−0.753 to −0.468	<0.001^a^
Antibiotic therapy (yes)	−0.006	−0.262 to 0.286	0.958
Systemic glucocorticoid therapy (yes)	−0.112	−0.312 to 0.108	0.344
Nebulized bronchodilator therapy (yes)	0.151	0.123 to 0.283	0.202
Comorbidity (yes)	−0.052	−0.269 to 0.180	0.665
Left ventricular ejection fraction (%)	0.080	−0.131 to 0.314	0.343

^a^Statistical significance.

BMI, body mass index; CAT, Chronic Obstructive Pulmonary Disease Assessment Test; mMRC, modified Medical Research Council dyspnea scale; EXACT, EXAcerbation of chronic pulmonary disease Tool; SGRQ, St George’s Respiratory Questionnaire; PaCO_2_, partial pressure of carbon dioxide in artery; FVC, forced vital capacity; FEV_1_, forced expiratory volume in the first 1 s; FEV_1_% predicted, the percentage of predicted forced expiratory volume in 1 s; FVC % predicted, the percentage of predicted forced vital capacity; PEF, peak expiratory flow; FEF_25–75%_, forced expiratory flow at 25 to 75% of the FVC; %LAA_−950_, the ratio of low attenuation areas below −950 HU to total lung volume measured by CT scan.

The four HRQoL questionnaires scores were significantly positively correlated with %LAA_−950_, and SGRQ scores were relatively high (*ρ* = 0.399, *p* < 0.001) (Figure 2). The indicators of postbronchodilator lung function were significantly negatively correlated with %LAA_−950_. The ratio of the forced expiratory volume in the first 1 s to the forced vital capacity of the lungs (FEV_1_/FVC) and FEF_25%–75%_ were strongly correlated with %LAA_−950_ (*ρ* = −0.735, *p* < 0.001, and *ρ* = −0.639, *p* < 0.001, respectively) (Table 3 and Figure 2). Among these laboratory parameters, the serum albumin levels and lymphocyte counts were significantly negatively correlated with %LAA_−950_ (*ρ* = −0.292, *p* = 0.012, and *ρ* = −0.370, *p* = 0.001, respectively). Further, the partial pressure of carbon dioxide (PaCO_2_) levels were significantly positively correlated with %LAA_−950_ (*ρ* = 0.330, *p* = 0.004). Meanwhile, the serum CRP levels and neutrophil and eosinophil counts were not correlated with %LAA_−950_.

To reduce the risk of type I errors in the correlational analyses of multiple variables, the Bonferroni correction was used to redetermine a stricter *α* level of 0.0020 (0.05/25) for re-evaluating the correlation between %LAA_−950_ and other parameters. Under the new test level, %LAA_−950_ was significantly correlated with BMI, mMRC grade, EXACT score, SGRQ score, lymphocyte counts, and lung function parameters (except FVC % predicted).

### Stepwise multiple regression analysis

A stepwise multiple linear regression analysis was conducted to determine emphysema predictors. Age, BMI, presence of chronic bronchitis, CAT score, mMRC grade, EXACT score, SGRQ score, lymphocyte counts, PaCO_2_ levels, albumin levels, FVC, the percentage of predicted forced vital capacity (FVC % predicted), FEV_1_, FEV_1_% predicted, FEV_1_/FVC, peak expiratory flow (PEF), and FEF_25–75%_ were included as covariates in the model. As shown in Table 4, in the final regression model, FEV_1_/FVC and age were found to be significant factors (*R*^2^ = 0.592; *F* = 50.762; *p* < 0.001).

**Table 4:** Stepwise multiple linear regression analysis for%LAA_−950_^a^.

Variables	Coefficient (95%CI)	Standardized *β*	*T*-value	*P*-value	VIF
Intercept	33.516 (11.223 to 55.810)		2.998	0.004	
FEV_1_/FVC	−0.944 (−1.140 to −0.747)	−0.732	−9.580	<0.001^b^	1.000
Age	0.443 (0.148 to 0.739)	0.228	2.990	0.004^b^	1.000

^a^*R*^2^ = 0.592; *F* = 50.762; p < 0.001; ^b^Statistical significance; variables: Age, BMI, presence of chronic bronchitis, CAT score, mMRC grade, EXACT score, SGRQ score, lymphocyte counts, PaCO_2_ levels, albumin levels, FVC, FVC % predicted, FEV_1_, FEV_1_% predicted, FEV_1_/FVC, PEF, and FEF_25%–75%._

%LAA_−950_, the ratio of low attenuation areas below −950 HU to total lung volume measured by CT scan; β, regression coefficient; VIF, variance inflation factor; BMI, body mass index; CAT, Chronic Obstructive Pulmonary Disease Assessment Test; mMRC, modified Medical Research Council dyspnea scale; EXACT, EXAcerbation of chronic pulmonary disease Tool; SGRQ, St George’s Respiratory Questionnaire; PaCO_2_, partial pressure of carbon dioxide in artery; FVC, forced vital capacity; FEV_1_, forced expiratory volume in the first 1 s; FEV_1_% predicted, the percentage of predicted forced expiratory volume in 1 s; FVC % predicted, the percentage of predicted forced vital capacity; PEF, peak expiratory flow; FEF_25–75%_, forced expiratory flow at 25 to 75% of the FVC.

## Discussion

This study investigated the correlation between CT-determined %LAA_−950_ and indicators, including lung function parameters, HRQoL assessment scores, and laboratory test results, which reflect AECOPD severity. Results found that %LAA_−950_ was associated with decreased lung function and poor HRQoL in inpatients with AECOPD. Therefore, CT-determined emphysema could be a potential indicator of AECOPD severity.

Our finding may be explained by two potential mechanisms. Emphysema is one of the fundamental pathological changes in COPD, driving expiratory flow obstruction (EFO). In patients with COPD, hyperinflation results from the loss of elastic recoil and the presence of EFO (1). Consequently, hyperinflation and EFO are closely related to emphysema. Nevertheless, key differences exist between these entities: emphysema represents structural destruction and is typically assessed using imaging techniques (20), whereas hyperinflation and EFO are the pathophysiological changes usually evaluated by functional residual capacity (FRC) and forced expiratory volume in 1 second (FEV₁) (21). However, EFO can also be reflected by expiratory-phase CT metrics, specifically %LAA_−856_ (the percentage of low attenuation area below −856 HU), which is highly correlated with inspiratory-phase emphysema (%LAA_−950_) in patients with stable COPD (22). During exacerbations, airway resistance increases abruptly due to bronchospasm, mucosal oedema, and sputum inspissation, which in turn worsens both EFO and hyperinflation (23). A moderate acute exacerbation of COPD is characterized by worsening expiratory airflow obstruction and increased lung hyperinflation (21). Taken together, one possible mechanism is that the severity of emphysema inferred from inspiratory CT reflects the degree of EFO and hyperinflation, which are known to worsen during acute exacerbations. Another possible mechanism is suggested by previous literature, which have identified the severity of emphysema as an independent predictor of frequent exacerbations in COPD patients (9). Thus, patients with more severe emphysema may be predisposed to greater illness severity once an acute exacerbation occurs.

Previous studies have mainly focused on emphysema in stable COPD (24), rather than acute exacerbation of the disease. The Global Initiative for Chronic Obstructive Lung Disease (GOLD) recommends that clinical variables (including symptoms, arterial blood gas parameters, CRP levels, and lung function) should be used for severity assessment (1). Symptom assessments are based on the patients’ responses to the questionnaires. Thus, the results are subjective indicators. Qualified lung function test results are frequently not obtained during acute exacerbation. Meanwhile, CT scan parameters are an objective indicator, and most patients can complete this noninvasive examination. Therefore, CT-determined emphysema can be a variable for evaluating AECOPD severity.

In stable COPD (with GOLD grades 3–4), the quantitative CT scan inspiratory or expiratory parameters of emphysema were significantly correlated with lung function indicators, such as TLC, FVC, RV, and FEV_1_/FVC (25). In the current study, the correlation coefficients (*ρ*) of %LAA_−950_ and FEV_1_% predicted and FEV_1_/FVC were −0.602 and −0.735, respectively. These values were higher than those of stable COPD (−0.490 and −0.610) (24). The stronger correlation may be attributed to decreased lung function during acute exacerbation (10).

Previous studies have shown that inspiratory emphysema parameters were positively correlated with the SGRQ score in stable COPD. However, they were not significantly correlated with the CAT score (25). Based on our results, CT-determined emphysema was significantly positively correlated with the SGRQ score, EXACT score, mMRC grade, and CAT score, which are widely used to assess the symptom burden of COPD (1). This finding indicated that CT-determined emphysema could partly reflect AECOPD severity. The possible explanations are as follows: During acute exacerbation, air trapping is aggravated (1), or patients with a high %LAA_−950_ have a greater symptom burden.

As mentioned in the previous text, previous studies mainly explored the correlation between emphysema and lung function parameters (24). Our study found that age, presence of chronic bronchitis, and arterial PaCO_2_ were positively correlated with %LAA_−950_. The correlation between advanced age and COPD is well understood. Aging results in enlargement of the alveolar spaces and loss of lung elasticity, known as senile emphysema (26), which accelerates the decline of pulmonary function. In the current study, univariate and multivariate analyses found that age was a predictor of emphysema. Luminal plugging in large airways is associated with the emphysema phenotype (15), and airway mucus hypersecretion is more likely to result in air trapping (27).

In this study, %LAA_−950_ was negatively correlated with BMI, lymphocyte counts, and serum albumin levels, which are indicators of malnutrition (28). Malnutrition is significantly and independently associated with disease severity and is a predictor of poor prognosis in COPD (1). Reduced skeletal muscle and abdominal fat leads to an increased thoracic cavity volume. Skeletal muscle dysfunction and ventilation inefficiency result in increased daily energy consumption (1). These could be the mechanisms related to the close correlation between malnutrition and emphysema in stable COPD. During acute exacerbation, patients frequently experience poor appetite and increased energy consumption (1) and impaired lymphocyte immunity (29), leading to more significant abnormalities of the above mentioned indicators. Furthermore, comorbidities are associated with lower survival and poorer quality of life in COPD patients (30). However, we found no significant correlation between the presence of comorbidities and %LAA_−950_. One potential explanation for this null finding is that comorbidities were not systematically or prospectively diagnosed in our study, which may have limited our ability to detect a true association.

SGRQ (31), EXACT (31), and CAT (10) are widely applied to evaluate the symptom burden of COPD and are validated tools for assessing the severity of acute exacerbations. We found that SGRQ, EXACT, and CAT were positively correlated with %LAA_−950_, thereby indicating that emphysema can help define the severity of exacerbations. HRQoL questionnaires are noninvasive and reproducible methods and have significant advantages in COPD assessment. Meanwhile the assessment requires patients to understand and answer the questionnaire items. CT-determined emphysema could be an objective assessment method for exacerbation, particularly in patients who cannot comprehend or complete the questionnaires.

The current study had several limitations. First, although cigarette-smoke exposure is the leading cause of emphysema (1), our results did not show a correlation between the amount of smoking and %LAA_−950._ The possible explanations for these contrasting results are as follows: the data used were from a secondary analysis, and the study was designed to explore luminal mucus in large airways rather than emphysema. This resulted in the existence of selection bias. Second, given the cross-sectional design, our study can only demonstrate a correlation and did not systematically collect stable-state lung function or emphysema data prior to the exacerbation episode; therefore, whether emphysema or delta FEV_1_ between basal and exacerbation states can serve as a predictor of AECOPD severity requires a prospective cohort study for further investigation. Third, our study did not systematically document infectious or non-infectious phenotypes to investigate their potential impact on emphysema, and further research is therefore warranted to address this critical issue. Finally, this study included patients with severe AECOPD who required hospitalization. Therefore, future studies are needed to validate its external authenticity in patients with mild and moderate exacerbations.

## Conclusion

CT-determined emphysema is associated with decreased lung function and poor HRQoL in patients with severe AECOPD. If the results can be replicated, our findings will show that %LAA_−950_ may be a potential indicator of AECOPD severity.

## Data Availability

The raw data supporting the conclusions of this article will be made available by the authors, without undue reservation.
